# Supplementary material for: A Critical View of the Application of the APEX Software (Aqueous Photochemistry of Environmentally-Occurring Xenobiotics) to Predict Photoreaction Kinetics in Surface Freshwaters
Source: Molecules. 2019 Dec 18;25(1):9. doi: 10.3390/molecules25010009 (PMC7017383; doi:10.3390/molecules25010009)
Supplement: Supplementary file 1 [file molecules-25-00009-s001.zip › 3.2.4_gcc-4.4.0.zip!/3.2.4_gcc-4.4.0/3.2.4_gcc-4.4.0/doc/octave/html/interpreter/Inheritance-and-Aggregation.html]

Inheritance and Aggregation - Untitled


Previous: Overloading Objects,
Up: Object Oriented Programming


---

### 33.5 Inheritance and Aggregation

Using classes to build new classes is supported by octave through the
use of both inheritance and aggregation.

Class inheritance is provided by octave using the `class`
function in the class constructor. As in the case of the polynomial
class, the octave programmer will create a struct that contains the
data fields required by the class, and then call the class function to
indicate that an object is to be created from the struct. Creating a
child of an existing object is done by creating an object of the
parent class and providing that object as the third argument of the
class function.

This is easily demonstrated by example. Suppose the programmer needs
an FIR filter, i.e. a filter with a numerator polynomial but a unity
denominator polynomial. In traditional octave programming, this would
be performed as follows.

```
     octave:1> x = [some data vector];
     octave:2> n = [some coefficient vector];
     octave:3> y = filter (n, 1, x);
```

The equivalent class could be implemented in a class directory
@FIRfilter that is on the octave path. The constructor is a file
FIRfilter.m in the class directory.

```
```
     ## -*- texinfo -*-
     ## @deftypefn {Function File} {} FIRfilter ()
     ## @deftypefnx {Function File} {} FIRfilter (@var{p})
     ## Creates an FIR filter with polynomial @var{p} as
     ## coefficient vector.
     ##
     ## @end deftypefn
     
     function f = FIRfilter (p)
     
       f.polynomial = [];
       if (nargin == 0)
         p = @polynomial ([1]);
       elseif (nargin == 1)
         if (!isa (p, "polynomial"))
           error ("FIRfilter: expecting polynomial as input argument");
         endif
       else
         print_usage ();
       endif
       f = class (f, "FIRfilter", p);
     endfunction
```
```

As before, the leading comments provide command-line documentation for
the class constructor. This constructor is very similar to the
polynomial class constructor, except that we pass a polynomial object
as the third argument to the class function, telling octave that the
FIRfilter class will be derived from the polynomial class. Our FIR
filter does not have any data fields, but we must provide a struct to
the `class` function. The `class` function will add an
element named polynomial to the object struct, so we simply add a
dummy element named polynomial as the first line of the constructor.
This dummy element will be overwritten by the class function.

Note further that all our examples provide for the case in which no
arguments are supplied. This is important since octave will call the
constructor with no arguments when loading ojects from save files to
determine the inheritance structure.

A class may be a child of more than one class (see the documentation
for the `class` function), and inheritance may be nested. There
is no limitation to the number of parents or the level of nesting
other than memory or other physical issues.

As before, we need a `display` method. A simple example might be

```
```
     function display (f)
     
       display(f.polynomial);
     
     endfunction
```
```

Note that we have used the polynomial field of the struct to display
the filter coefficients.

Once we have the class constructor and display method, we may create
an object by calling the class constructor. We may also check the
class type and examine the underlying structure.

```
     octave:1> f=FIRfilter(polynomial([1 1 1]/3))
     f.polynomial = 0.333333 + 0.333333 * X + 0.333333 * X ^ 2
     octave:2> class(f)
     ans = FIRfilter
     octave:3> isa(f,"FIRfilter")
     ans =  1
     octave:4> isa(f,"polynomial")
     ans =  1
     octave:5> struct(f)
     ans =
     {
     polynomial = 0.333333 + 0.333333 * X + 0.333333 * X ^ 2
     }
```

We only need to define a method to actually process data with our
filter and our class is usable. It is also useful to provide a means
of changing the data stored in the class. Since the fields in the
underlying struct are private by default, we could provide a mechanism
to access the fields. The `subsref` method may be used for both.

```
```
     function out = subsref (f, x)
       switch x.type
         case "()"
           n = f.polynomial;
           out = filter(n.poly, 1, x.subs{1});
         case "."
           fld = x.subs;
           if (strcmp (fld, "polynomial"))
             out = f.polynomial;
           else
             error ("@FIRfilter/subsref: invalid property \"%s\"", fld);
           endif
         otherwise
           error ("@FIRfilter/subsref: invalid subscript type for FIR filter");
       endswitch
     endfunction
```
```

The "()" case allows us to filter data using the polynomial provided
to the constructor.

```
     octave:2> f=FIRfilter(polynomial([1 1 1]/3));
     octave:3> x=ones(5,1);
     octave:4> y=f(x)
     y =
     
        0.33333
        0.66667
        1.00000
        1.00000
        1.00000
```

The "." case allows us to view the contents of the polynomial field.

```
     octave:1> f=FIRfilter(polynomial([1 1 1]/3));
     octave:2> f.polynomial
     ans = 0.333333 + 0.333333 * X + 0.333333 * X ^ 2
```

In order to change the contents of the object, we need to define a
`subsasgn` method. For example, we may make the polynomial field
publicly writeable.

```
```
     function out = subsasgn (f, index, val)
       switch (index.type)
         case "."
           fld = index.subs;
           if (strcmp (fld, "polynomial"))
             out = f;
             out.polynomial = val;
           else
             error ("@FIRfilter/subsref: invalid property \"%s\"", fld);
           endif
         otherwise
           error ("FIRfilter/subsagn: Invalid index type")
       endswitch
     endfunction
```
```

So that

```
     octave:6> f=FIRfilter();
     octave:7> f.polynomial = polynomial([1 2 3]);
     f.polynomial = 1 + 2 * X + 3 * X ^ 2
```

Defining the FIRfilter class as a child of the polynomial class
implies that and FIRfilter object may be used any place that a
polynomial may be used. This is not a normal use of a filter, so that
aggregation may be a more sensible design approach. In this case, the
polynomial is simply a field in the class structure. A class
constructor for this case might be

```
```
     ## -*- texinfo -*-
     ## @deftypefn {Function File} {} FIRfilter ()
     ## @deftypefnx {Function File} {} FIRfilter (@var{p})
     ## Creates an FIR filter with polynomial @var{p} as
     ## coefficient vector.
     ##
     ## @end deftypefn
     
     function f = FIRfilter (p)
     
       if (nargin == 0)
         f.polynomial = @polynomial ([1]);
       elseif (nargin == 1)
         if (isa (p, "polynomial"))
           f.polynomial = p;
         else
           error ("FIRfilter: expecting polynomial as input argument");
         endif
       else
         print_usage ();
       endif
       f = class (f, "FIRfilter");
     endfunction
```
```

For our example, the remaining class methods remain unchanged.
